# Optimal proteome allocation strategies for phototrophic growth in a light-limited chemostat

**DOI:** 10.1186/s12934-019-1209-7

**Published:** 2019-10-10

**Authors:** Marjan Faizi, Ralf Steuer

**Affiliations:** Institut für Biologie, Fachinstitut für Theoretische Biologie, Humboldt-Universität zu Berlin, Invalidenstr. 110, 10115 Berlin, Germany

**Keywords:** Cyanobacteria, Microalgae, Photobioreactor, Biofuels, Photosynthetic productivity, Resource allocation, *Synechocystis* sp. PCC 6803

## Abstract

**Background:**

Cyanobacteria and other phototrophic microorganisms allow to couple the light-driven assimilation of atmospheric $\text {CO}_{2}$ directly to the synthesis of carbon-based products, and are therefore attractive platforms for microbial cell factories. While most current engineering efforts are performed using small-scale laboratory cultivation, the economic viability of phototrophic cultivation also crucially depends on photobioreactor design and culture parameters, such as the maximal areal and volumetric productivities. Based on recent insights into the cyanobacterial cell physiology and the resulting computational models of cyanobacterial growth, the aim of this study is to investigate the limits of cyanobacterial productivity in continuous culture with light as the limiting nutrient.

**Results:**

We integrate a coarse-grained model of cyanobacterial growth into a light-limited chemostat and its heterogeneous light gradient induced by self-shading of cells. We show that phototrophic growth in the light-limited chemostat can be described using the concept of an average light intensity. Different from previous models based on phenomenological growth equations, our model provides a mechanistic link between intracellular protein allocation, population growth and the resulting reactor productivity. Our computational framework thereby provides a novel approach to investigate and predict the maximal productivity of phototrophic cultivation, and identifies optimal proteome allocation strategies for developing maximally productive strains.

**Conclusions:**

Our results have implications for efficient phototrophic cultivation and the design of maximally productive phototrophic cell factories. The model predicts that the use of dense cultures in well-mixed photobioreactors with short light-paths acts as an effective light dilution mechanism and alleviates the detrimental effects of photoinhibition even under very high light intensities. We recover the well-known trade-offs between a reduced light-harvesting apparatus and increased population density. Our results are discussed in the context of recent experimental efforts to increase the yield of phototrophic cultivation.

## Background

Phototrophic microorganisms such as microalgae and cyanobacteria hold significant potential for the production of industrially or medically relevant compounds, such as pigments, organic acids, or alcohols [17, 56], as well as secondary metabolites used for pharmaceutical purposes [26, 36]. The interest in cyanobacteria as platforms for microbial cell factories originates from their capability for carbon-neutral production, easy accessibility for genetic manipulation, and their relatively fast growth rates compared to land plants. A major challenge of cultivating phototrophic microorganisms on a commercial scale, however, is still the low biomass density, and hence low volumetric productivity, compared to other biotechnologically relevant microorganisms [27, 29, 51, 52].

Previous research has established the critical role of the photobioreactor design for improving the overall performance with a focus on parameters such as mixing rates, gas exchanges, temperature, pH, as well as light paths [22, 40, 41]. In particular, there has been significant progress to model phototrophic culture systems making use of sophisticated computational methods to describe reactor geometry, light transfer, and fluid dynamics [1, 9, 16, 38]. Concomitantly, there have been significant efforts to obtain a better quantitative understanding of the photosynthetic productivity of cyanobacterial growth in photobioreactors [8, 43].

However, despite this progress, there remains a need for an improved computational framework to better understand the physiological acclimation of cyanobacteria in a heterogeneous light environment typically encountered in dense cultures. In this respect, we can build upon an established theory of the light-limited chemostat, originally developed by Huisman et al. [23] and later refined by other authors [18, 30, 31]. These previous analyses, however, were almost all based on phenomenological growth models, such as the Monod or Haldane-type equation, and only few works, such as the computational analysis of He et al. [20], explicitly integrate intra- and extracellular information to achieve a better understanding of bioreactor productivities.

The purpose of this work is therefore to integrate a recent coarse-grained model of cyanobacterial growth into a model of population dynamics within a light-limited chemostat. The coarse-grained computational model was previously parametrized using an in-depth quantitative analysis of cyanobacterial growth in an optically thin turbidostat [54], and describes the relationship between intracellular protein allocation and cellular growth. Based on our previous experimental analyses [15, 54], our premise is that the model provides a reasonable description of cyanobacterial growth under different light intensities—and therefore represents a suitable starting point to investigate the relationship between the allocation of intracellular proteins, light absorption, self-shading, growth rate, and overall culture productivity. Combining our model of cyanobacterial growth with a model of a light-limited chemostat therefore allows us to computationally investigate and compare different possible proteome allocation strategies, such as maximization of growth rate versus maximization of culture productivity, and provides insights into optimal strain design strategies.

Our results have profound consequences for the design of photobioreactors. The model predicts that high population densities alleviate the detrimental effects of photoinhibition even under very high light intensities. The results therefore strongly support previous works by Richmond [41] and Qiang et al. [40] who showed that a high areal phototrophic productivity can be achieved using reactors maximally exposed to light with a short light-path and turbulent mixing. We further recover the well-known trade-offs between a reduced light-harvesting apparatus and increased population density, and hence higher volumetric productivity. Our approach provides a general computational framework to integrate and solve models of cyanobacterial proteome allocation in a light-limited chemostat.

The paper is organized as follows: in the first two sections, we briefly introduce a model of the light-limited chemostat. In the subsequent sections, we describe the coarse-grained model of phototrophic growth and its solution using the assumption of parsimonious protein allocation. We then integrate both models and show that, as a nontrivial result, phototrophic growth in a light-limited chemostat can be described using the concept of an average light intensity. We then investigate culture properties, such as light attenuation, population density, and volumetric productivity, as well as the emergent bistability of the culture induced by photoinhibition. In the subsequent section, we consider hypothetical strains whose protein allocation is optimized for maximal culture productivity—and highlight differences to proteome allocation in wild-type cells. Finally, we consider engineering strategies for heterologous production.

## Results

### A model of the light-limited chemostat

**Fig. 1:**
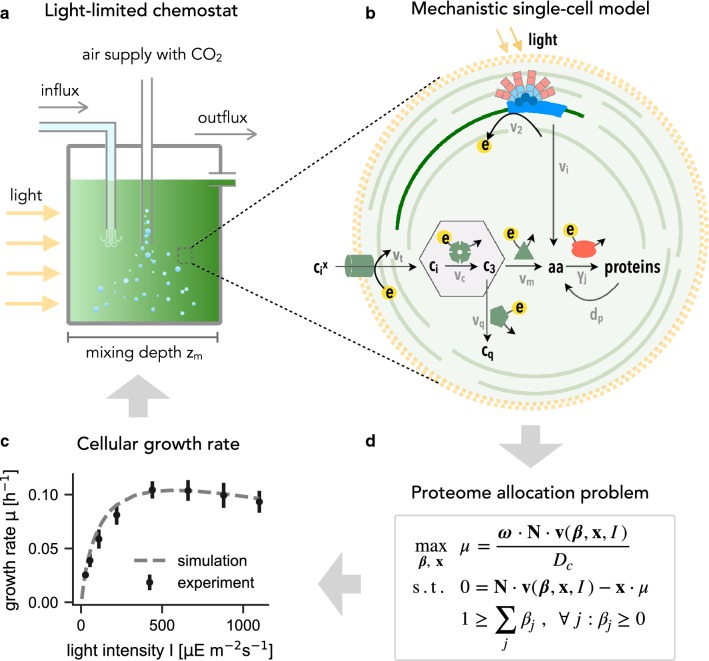
A model of the light-limited chemostat. **a** Schematic representation. The light source irradiates a culture vessel of depth $z_m$. The culture is aerated with $\text {CO}_{2}$-enriched air and nutrients are well mixed. Different from other nutrients, the photon flux is inhomogeneous and decays exponentially with depth. **b** A coarse-grained single-cell model. The model describes carbon assimilation and metabolism, light harvesting, photosynthesis, and protein translation by ribosomes. External inorganic carbon $c_i^x$ is transported into the cell ($v_t$), assimilated ($v_c$) into organic carbon precursors $c_3$ from which amino acids *aa* are synthesized ($v_m$). Amino acids serve as precursors for protein synthesis ($\gamma _j$). The model consists of seven coarse-grained protein complexes, including ribosomes *R*, transport proteins $E_T$, metabolic enzymes $E_C$, $E_M$, $E_Q$, photosynthetic units *PSU*, and quota proteins $P_Q$. All catalyzed reactions are fueled by energy units *e* that are produced by activated photosynthetic units $PSU^{*}$ ($v_2$). Activation of resting photosynthetic units $PSU^0$ is facilitated by light. High light intensities cause photodamage ($v_i$), i.e., the degradation of PSU into its constituent amino acids. The model also incorporates a general protein degradation term ($d_p$), as well as an energy maintenance reaction ($v_{me}$). **c** The optimized specific growth rate as a function of light intensity, shown together with experimental values for *Synechocystis* sp. PCC 6803 obtained from quantitative growth experiments in an optically thin turbidostat [15, 54]. **d** Proteome allocation within the system is formulated as an optimization problem (parsimonious allocation) such that the ribosome fractions $\beta _j$ translating the different proteins give rise to a maximal specific growth rate $\mu$

To investigate cellular proteome allocation in dense cultures, we make use of a mathematical description of continuous cultivation in a chemostat, as originally described by Novick and Szilard [37] and, independently, by Monod [35]. The dynamics of the population density $\varrho$ (in units of cells per milliliter) of genetically identical and well mixed cells is described by the differential equation,

1 $$\begin{aligned} \frac{d\varrho }{dt} = \mu \cdot \varrho - D \cdot \varrho, \end{aligned}$$

where $\mu$ denotes the specific cellular growth rate and *D* denotes the dilution rate of the culture medium. Figure 1 illustrates the concept of the chemostat.

Fresh medium and dissolved nutrients are continuously fed into the culture with the same rate as the culture medium is removed, resulting in a constant operating volume. All dissolved nutrients are well mixed within the culture medium. The dynamics of the concentration of a nutrient *s* depends on the inflow and outflow rates, as well as the uptake rate of the microorganisms,

2 $$\begin{aligned} \frac{d[s]}{dt} = V_{in,s} - D \cdot [s] - \frac{1}{Y_s} \cdot \mu \cdot \varrho, \end{aligned}$$

where $V_{in,s}$ denotes the inflow rate of the nutrient *s*, with $V_{in,s} = [s_0] \cdot D$ for a soluble nutrient that is supplied with a concentration $[s_0]$ via the medium. Gaseous nutrients, such as $\text {CO}_{2}$, are supplied by sparging. The uptake rate of nutrients by the microorganisms is typically assumed to be proportional to the specific growth rate $\mu$ and the yield coefficient $Y_s$ denoting the number of cells obtained per nutrient molecule.

### Light attenuation in the chemostat

Different to other potentially limiting nutrients, light cannot be homogeneously distributed through the culture by vigorous mixing. Following Huisman et al. [23] and others [4, 18, 20, 30, 31], we describe light absorption according to the law of Lambert–Beer, i.e., we assume that light absorption is proportional to the concentration of light-absorbing substances in the medium (including cells) and the local light intensity. The light intensity *I*(*z*) at a depth *z* is then given by

3 $$\begin{aligned} I(z) = I_0 \cdot e^{- ( \alpha \cdot \varrho + K_{bg}) \cdot z }, \end{aligned}$$

where $I_0$ denotes the incident light intensity at the surface, $\alpha$ denotes the species-specific light attenuation coefficient per cell, and $K_{bg}$ denotes the background turbidity of the medium including all other light-absorbing substances [23]. We note that Lambert-Beer’s law is an approximation and neglects aspects such as backscattering. In the following, we further assume monochromatic light and consider light as the only limiting nutrient. The latter assumption is motivated by the fact that in biotechnological applications mineral nutrients are typically supplied in sufficient quantities. Suitable strategies to supply $\text {CO}_{2}$ to dense cultures were recently proposed [3, 29].

To solve the equations for the light-limited chemostat requires knowledge of the specific growth rate $\mu (I)$ as a function of the light intensity (and possibly other nutrients). To this end, previous works typically used well-known phenomenological rate equations, such as the Monod equation in Huisman et al. [23], to describe the light-limited growth of phototrophic microorganisms. Following the original analysis of Huisman et al. [23], Gerla et al. [18] and later Martínez et al [31] provided a detailed analysis based on a Haldane-type equation,

4 $$\begin{aligned} \mu (I) = \frac{\mu _{max} \cdot I}{k_1 + I + k_2 \cdot I^2} \ , \end{aligned}$$

where $k_1$ and $k_2$ denote species-specific parameters and $\mu _{max}$ the maximal growth rate in the absence of photoinhibition ($k_2=0$). Equation (4) can be derived using a simple model of photoinhibition [14, 19]. See, for example, Westermark and Steuer [50] for a review.

### A coarse-grained model of phototrophic growth

Our aim is to replace the phenomenological growth equations used in previous works with a mechanistic model of cyanobacterial growth. To this end, we utilize the coarse-grained model of Faizi et al. [15] with minor modifications as described in “Methods” section. The model describes phototrophic growth of a single cyanobacterial cell in an optically thin culture and was recently subject to an in-depth analysis based on quantitative growth experiments [54]. Different to phenomenological growth models, the model of Faizi et al. [15] describes growth in terms of the expression of a (coarse-grained) proteome and accounts for the acclimation of cells to different light intensities. Based on our previous experimental analysis [54], we consider the model to be a reasonable description of cyanobacterial growth, and therefore a suitable starting point to investigate growth in a light-limited chemostat.

The model is conceptually similar to other recent models of cellular resource allocation [5, 24, 34, 49] and describes the uptake and conversion of extracellular nutrients into metabolic precursors (metabolism) as well as the synthesis of proteins from these metabolic precursors (gene expression). The dynamics of all cellular constituents are modelled as ordinary differential equations (ODEs). In brief, the model consists of 13 ODEs that describe the dynamics of 7 intracellular protein complexes and 5 intracellular metabolites: inorganic carbon $c_i$ is taken up and assimilated into the metabolite $c_3$, which serves as a precursor for the synthesis of amino acids *aa* and other cellular components $c_q$. Proteins are translated by ribosomes using available amino acids and cellular energy. Energy is provided by a photosynthetic unit *PSU* that integrates light harvesting and the electron transport chain. The cellular energy unit *e* combines chemical energy and reductant (ATP and NADPH, respectively). Light absorption induces photodamage that results in a (light-dependent) degradation of *PSU* back into its constituent amino acids. The model is depicted in Fig. 1 and a detailed description of the model equations is provided in “Methods” section.

### Parsimonious resource allocation and growth

Similar to other models of cellular resource allocation, the model does not assume knowledge of regulatory interactions but is formulated as an optimization problem that is solved based on the principle of parsimonious allocation of cellular resources to achieve a maximal growth rate. That is, we maximize the cellular growth rate under (steady-state) balanced growth conditions by varying the fractions $\beta _j$ of ribosomes that translate specific proteins $P_j$. The fractions $\beta _j$ govern the abundance of the respective proteins—and a different allocation of intracellular proteins will give rise to different physiological properties and growth rates under different environmental conditions. Hence, our framework goes beyond phenomenological growth functions and allows us to study the consequences of different proteome allocation strategies, including allocation strategies that are optimized for maximal culture productivity, as well as the trade-offs that arise from an heterologous synthesis and excretion of a metabolic compound of interest.

Assuming steady-state conditions and balanced growth, all intracellular components are subject to the mass balance constraint [11, 15],

5 $$\begin{aligned} 0 = \mathbf{N} \cdot \mathbf{v}{({\varvec{\beta }}, \mathbf{x}, I)}- \mu \cdot \mathbf{x}, \end{aligned}$$

where $\mathbf{N}$ denotes the stoichiometric matrix, $\mathbf{v}$ the vector of reaction fluxes, and $\mathbf{x}$ the vector of intracellular concentrations (including proteins). Equation (5) implies that the product of the stoichiometric matrix $\mathbf{N}$ and the vector of reaction fluxes $\mathbf{v}$ equals the dilution of intracellular compounds due to growth. With ${\varvec{\omega }}$ denoting the vector of specific weights of each intracellular compound, and the (reasonable) assumption of a constant cell density $D_c = {\varvec{\omega }} \cdot \mathbf{x}$, the specific cellular growth rate is given by

6 $$\begin{aligned} \mu {({\varvec{\beta }}, \mathbf{x}, I)} = \frac{{\varvec{\omega }} \cdot \mathbf{N} \cdot \mathbf{v}{({\varvec{\beta }}, \mathbf{x}, I)}}{D_c}. \end{aligned}$$

Figure 1c shows the resulting maximal growth rate in dependence of the light intensity *I*. The growth curve emerges from the coarse-grained model using the assumption of parsimonious protein allocation and is in good agreement with Eq. (4), as well as with experimentally determined growth curves obtained in an optically thin turbidostat. See Faizi et al. [15] and Zavřel et al. [54] for further discussion.

### Phototrophic growth in the light-limited chemostat

To describe phototrophic growth in a light-limited chemostat, we aim to incorporate the coarse-grained growth model into a heterogeneous light environment induced by self-shading of the culture. According to Eq. (3), the local light intensity decreases exponentially as a function of vessel depth $z_m$ and depends on the density of light-absorbing organisms $\varrho$ and their species-specific light attenuation coefficient $\alpha$. Within our model the species-specific cellular light attenuation coefficient is given by

7 $$\begin{aligned} \alpha = \alpha _0 + \hat{\sigma } \cdot PSU^{tot}, \end{aligned}$$

where $\alpha _0$ denotes a basal light absorption per cell independent of photosynthesis, and the product $\hat{\sigma }\ \cdot \ PSU^{tot}$ describes the absorption per cell for photosynthesis, with $\hat{\sigma }$ denoting the effective cross section per PSU. The specific light attenuation coefficient $\alpha$ therefore depends on the expression of the protein complex PSU, and hence on the acclimation state of the cell.

We further assume that the culture is rapidly mixed, i.e., we only consider a single cell type and acclimation state within the culture. The concentrations of intracellular compounds do not depend on the (momentary) position of a cell within the chemostat. Individual metabolic reactions, however, in particular reactions that directly depend on light, will proceed with rates that depend on the local light intensity—the overall metabolism is required to be balanced with respect to energy uptake and growth. As noted by Pirt [39], this assumption implies a certain buffering capacity to permit each cell to grow with a constant rate even though it is intermittently exposed to radiation.

To solve the model, we consider the steady-state condition for the chemostat, Eq. (1), and integrate over the vessel depth $z_m$,

8 $$\begin{aligned} 0 = \frac{1}{z_m} \int _0^{z_m} \mu \cdot \varrho \cdot dz - D \cdot \varrho. \end{aligned}$$

Using the definition of the specific growth rate, Eq. (6), we obtain an expression for the effective growth rate $\hat{\mu }$ in the chemostat,

9 $$\begin{aligned} \hat{\mu } {({\varvec{\beta }}, \mathbf{x}, \hat{I})} = \frac{{\varvec{\omega }} \cdot \mathbf{N}}{D_c} \cdot \hat{\mathbf{v}} {({\varvec{\beta }}, \mathbf{x}, \hat{I})}, \end{aligned}$$

with

10 $$\begin{aligned} \hat{\mathbf{v}} {({\varvec{\beta }}, \mathbf{x}, \hat{I})} := \frac{1}{z_m} \int _0^{z_m} \mathbf{v} {({\varvec{\beta }}, \mathbf{x}, I(z))}\ \ dz. \end{aligned}$$

Using a substitution of variables, as suggested by Huisman et al. [23], Eq. (10) can be rewritten as an integral over light intensity and solved analytically for all reaction rates (see “Methods” section). The solution reveals that it is possible to express the effective specific growth rate $\hat{\mu }$ in the light-limited chemostat as a function of an effective average light intensity $\hat{I}$,

11 $$\begin{aligned} \hat{I} := \frac{I_0 - {I(z_m)}}{\ln (I_0) - \ln ({I(z_m)})}. \end{aligned}$$

Since $\ln (I_0) -\ln (I(z_m)) = ( \alpha \cdot \varrho + K_{bg}) \cdot z_m$, the average light intensity $\hat{I}$ depends on the incident light intensity $I_0$, the cell-specific attenuation coefficient $\alpha$, the background turbidity $K_{\mathrm{bg}}$, the population density $\varrho$ and the vessel depth $z_m$. As already noted by Huisman et al. [23], the value of $\hat{I}$ can also be readily estimated experimentally from measuring the incident and transmitted light intensities, $I_0$ and $I(z_m)$, respectively.

The solution of Eq. (10) is a nontrivial result and crucially depends on the assumption of rapid mixing and the fact that light absorption and photoinhibition are modelled as first-order reactions. There is indeed significant empirical evidence for the latter assumption, which is contrary to the belief that photoinhibition does not occur under low light [6, 44, 45]. While previous models also used the concept of an average light intensity as a convenient approximation [7, 13], the description emerges here as a consequence of the functional form of the intracellular rate equations.

### Growth, population density and bistability

Given the definitions above, a solution of the model of the light-limited chemostat requires to solve the steady-state equation $0=\hat{\mu } {({\varvec{\beta }}, \mathbf{x}, \hat{I})} \cdot \varrho - D \cdot \varrho$ for the effective growth rate $\hat{\mu }$ as a function of the average light intensity $\hat{I}$. A solution requires knowledge about the cellular proteome allocation, or, as described above, a suitable optimization objective. As our first optimization scenario, we therefore assume that the cyanobacterial cells acclimate to the average light intensity $\hat{I}$ only. That is, we assume that the cells have no explicit information about the culture density or other culture parameters, but adjust their intracellular proteome such that it maximizes the effective growth rate $\hat{\mu }$ for the respective average light intensity $\hat{I}$. This allocation strategy is identical to the proteome allocation strategy previously used in Faizi et al. [15], with results that are in excellent agreement with measurements in an optically thin turbidostat [54]. Our premise is therefore that the cyanobacterial wild-type (WT) strain has evolved to allocate its proteome such that the specific growth rate in the respective light environment is maximized. We denote this optimization objective as WT-strategy.

**Fig. 2:**
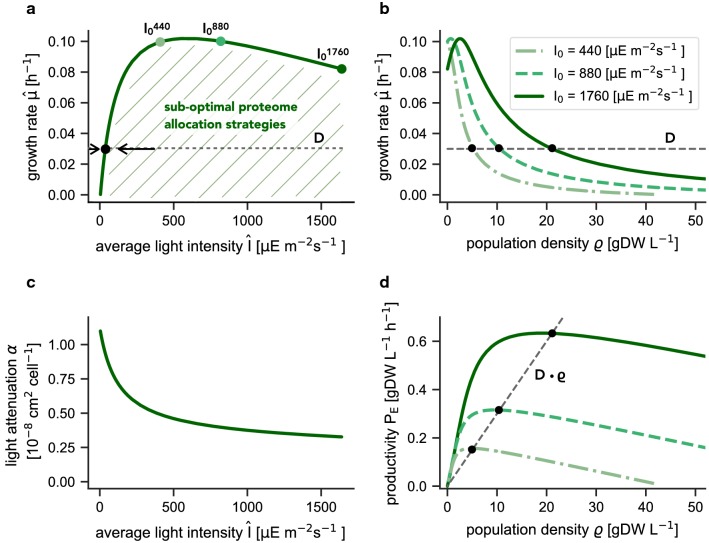
Properties of the light-limited chemostat. **a** The maximal effective growth rate $\hat{\mu }$ as a function of the average light intensity. The shaded area indicates sub-optimal proteome allocation strategies. The average light intensity $\hat{I}$ is bound from above by the (a function of the) incident light intensity $I_0$. The limits for three different incident light intensities are shown. A steady state is attained if the effective growth rate $\hat{\mu }$ equals the dilution rate. **b** The maximal effective growth rate $\hat{\mu }$ as a function of the population density for three different incident light intensities $I_0$. Higher incident light intensities result in a higher steady-state population density for an identical dilution rate *D*. For certain dilution rates, bistability emerges. **c** The cellular light attenuation coefficient $\alpha$ as a function of the average light intensity $\hat{I}$. Parsimonious allocation results in an acclimation of cells to different light intensities. **d** The culture productivity $P_E = \hat{\mu } \cdot \varrho$ as a function of the population density for three incident light intensities $I_0$ . At steady-state, the effective growth rate equals the dilution rate, hence $P_E = D \cdot \varrho$. Higher incident light intensities result in a higher steady-state productivity

Figure 2 illustrates the solution obtained for the light-limited chemostat for the WT-strategy. All extracellular culture parameters are summarized in Table 1. Figure 2a shows the maximal effective growth rate $\hat{\mu }$ as a function of the effective average light intensity $\hat{I}$. For any value of $\hat{I}$, sub-optimal proteome allocation strategies result in growth rates beneath the curve (indicated by the shaded area in Fig. 2a). The maximal value of the effective average light intensity $\hat{I}$ is bound from above by (a function of) the incident light intensity $I_0$. Figure 2a also indicates that the WT-strategy is (evolutionary) stable with respect to changes in proteome allocation: any sub-optimal proteome allocation strategy results in a lower growth rate for the respective average light intensity. The respective strain would be outcompeted by a strain that attains a higher specific growth rate at the same average light intensity. Figure 2b shows the effective growth rate $\hat{\mu }$ as a function of the population density. We note that the values are identical to those shown in Fig. 2a, $\hat{\mu }$ is not subject to any explicit optimization with respect to the population density $\varrho$.

**Table 1:** Extracellular parameters for the light-limited chemostat

Name	Definition	Value
$\text {c}_{\mathrm{i}}^{\mathrm{x}}$	External inorganic carbon	100 mM
$\text {z}_{\mathrm{m}}$	Depth of culture vessel	2.4 cm
$\text {K}_{\mathrm{bg}}$	Background turbidity	$0.06\ \text {cm}^{-1}$
$\alpha _{0}$	Basal light attenuation	$0.01\ \upmu \text {m}^{2}\ \text {cell}^{-1}$

For definitions and derivations see “Methods” section

Different to phenomenological models, the assumption of parsimonious protein allocation implies that cells acclimate to different average light intensities $\hat{I}$. The respective changes in the cellular light attenuation coefficient $\alpha$ are shown in Fig. 2c. Higher average light intensities result in the lower expression of photosynthetic units, resulting in lower values of the cellular light attenuation coefficient $\alpha$. Figure 2d shows the culture productivity $P_E = \hat{\mu } \cdot \varrho$ as a function of the population density for different incident light intensities $I_0$. The steady-state productivity is given by the intersection between the curve and the straight line defined by $D \cdot \varrho$. The culture productivity $P_E$ has a maximum for intermediate values of *D* that depends on the incident light intensity $I_0$.

The chemostat is in steady state when the effective growth rate $\hat{\mu }$ equals the dilution rate *D*. For any incident light intensity $I_0$, we must therefore distinguish between three possible cases (see Fig. 2a, b): (i) For a sufficiently low dilution rate *D*, there is a single steady state. In this case, the average light intensity $\hat{I}$ corresponds to the nutrient concentration in the classical chemostat. Any perturbation towards a higher culture density reduces the average light intensity, resulting in a lower growth rate and hence a decreasing culture density: the steady state is stable. (ii) For a dilution rate *D* that exceeds the maximal growth rate $\hat{\mu }^{\mathrm{max}}$, no positive steady state is feasible, the culture is washed out ($\varrho =0$). (iii) For intermediate values of *D*, and for sufficiently high $I_0$, the effects of photoinhibition induce a second potential steady state: the chemostat is bistable. In the second state, however, an increase in the average light intensity results in a decrease of the effective growth rate, resulting in a decrease of the population density, and hence a further increase in the resulting average light intensity: the second steady state is unstable and the culture is washed out (${\varrho }=0$).

These results recapitulate the results previously obtained for phenomenological rate equations. In particular, Gerla et al. [18] and others [30, 31] provided a detailed theoretical analysis of the light-limited chemostat using Haldane-type models and highlight the consequences of bistability induced by photoinhibition. In the following, we focus on the stable steady state only and make use of the plasticity of our model to investigate different proteome allocation strategies.

### Maximizing photosynthetic productivity

For many biotechnological applications the overall culture productivity is a crucial process parameter that determines the economic viability of phototrophic cultivation. We are therefore interested in the maximal volumetric productivity of a light-limited chemostat, as well as the optimal proteome allocation strategy to achieve maximal productivity—and how this strategy differs from proteome allocation in wild-type cells. To this end, we consider a hypothetical strain that is engineered (or selected) to adjust its intracellular protein allocation such that it maximizes the steady-state volumetric biomass productivity of the culture, defined as

12 $$\begin{aligned} P_E = \hat{\mu } \cdot \varrho. \end{aligned}$$

We denote this optimization objective as $\text {P}_{\mathrm{E}}$-strategy.

**Fig. 3:**
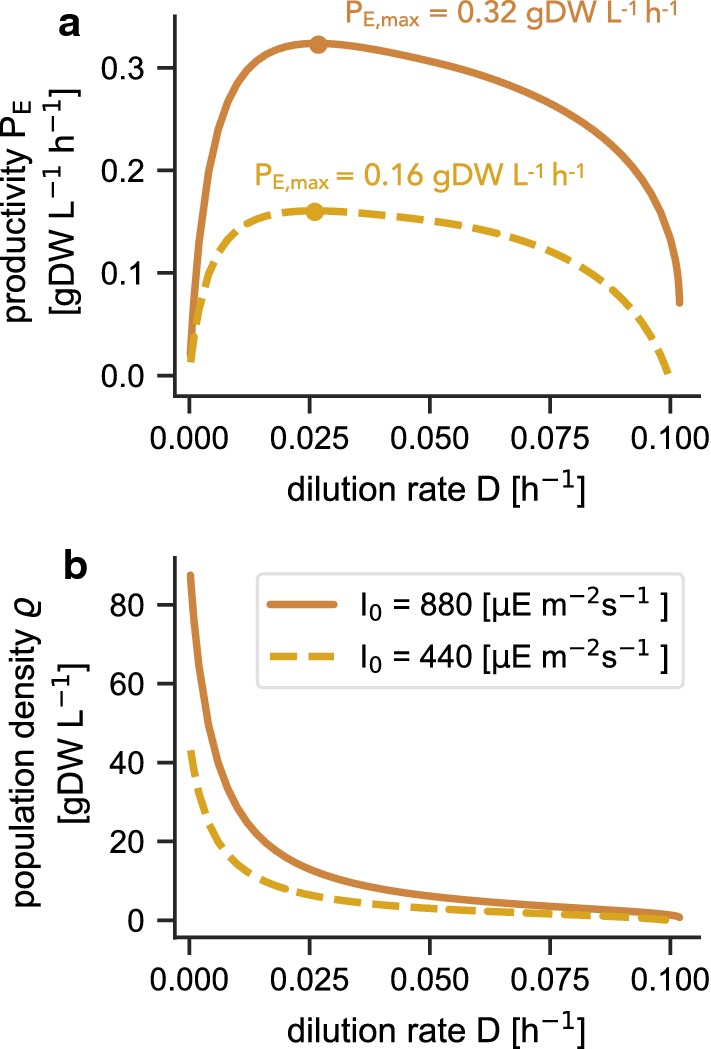
Maximizing photosynthetic productivity. **a** The maximal volumetric biomass productivity $P_E$ as a function of the dilution rate *D* for two different incident light intensities $I_0$. **b** The population density $\varrho$ as a function of *D*. The maximal productivity $P_E$ represents a trade-off between a high population density and a high dilution rate *D*, resulting in a maximal value for intermediate dilution rates, well below the maximal growth rate of the strain

Figure 3a shows the maximal biomass productivity of the hypothetical $\text {P}_{\mathrm{E}}$-strain as a function of the dilution rate *D* for two different incident light intensities $I_0$. The curves reflect a trade-off between maximizing the dilution rate and maximizing culture density $\varrho$. With increasing dilution rates (Fig. 3b) the steady-state population density decreases, resulting in a maximal biomass productivity $P_E$ for intermediate values of *D*. We note that the dilution rates at which the maximal productivity is attained are well below the maximal growth rate of the strain. For example, for an incident light intensity of $I_0=440\ \upmu \text {E}\ \text {m}^{-2}\ \text {s}^{-1}$, the maximal productivity $P_E=0.16\ \text {gDW L}^{-1}\ \text {h}^{-1}$ is achieved for a dilution rate $D_{\mathrm{opt}} = 0.026\ \text {h}^{-1}$.

**Fig. 4:**
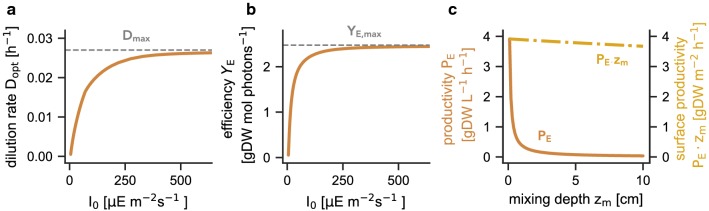
Photosynthetic efficiency, optimal dilution, and effects of the mixing depths. **a** The optimal dilution rate $D_{\mathrm{opt}}$ for which a maximal biomass productivity $P_E$ is attained. $D_{\mathrm{opt}}$ increases with increasing incident light intensity $I_0$ and saturates at a value $D_{max} \approx 0.027\ \text {h}^{-1}$. **b** The photosynthetic efficiency as a function of the incident light intensity for a maximally productive chemostat. The photosynthetic efficiency increases with increasing incident light intensity and saturates at $Y_{E,max} \approx 2.474\ \text {gDW mol photons}^{-1}$. **c** The consequences of different mixing depths on the maximal biomass productivity $P_E$. While the volumetric productivity decreases, the surface productivity $P_E \cdot z_m$ remains approximately constant. The slight decrease is due to the increasing effect of the background turbidity

Figure 4a shows the optimal dilution rate $D_{\mathrm{opt}}$ for different incident light intensities $I_0$. The dilution rate $D_{\mathrm{opt}}$ for which the maximal productivity is attained initially increases with the incident light intensity and converges to $D_{\mathrm{opt}} \approx 0.027\ \text {h}^{-1}$. Similar, as shown in Fig. 4b, the photosynthetic efficiency $Y_E$, defined as the photosynthetic yield in gDW per photon [57],

13 $$\begin{aligned} Y_E = \frac{P_E \cdot z_m}{I_0}, \end{aligned}$$

first increases with increasing incident light intensity and then saturates at $Y_E\ \approx \ 2.37\ \text {gDW mol photons}^{-1}$, approximately double that of the measured efficiencies from Touloupakis et al. [43] (see “Methods” section for a discussion of error ranges). These results suggest that the operation of a light-limited chemostat is more efficient at high light intensities and that the efficiency is also not decreasing for incident light intensities under which the individual cells already exhibit strong photoinhibition.

Finally, we consider the impact of the vessel depth $z_m$ on the (maximal) productivity. As expected, and shown in Fig. 4c, the culture density and hence the volumetric biomass productivity decreases with increasing vessel depth. However, the productivity per surface area (as well as the total biomass within the bioreactor) remains approximately constant for different vessel depths. The small decrease of the per surface area is due to the increasing effect of the background absorption [$K_{\mathrm{bg}}$ in Eq. (3)]. Hence, the model predictions agree with previous reports from Qiang et al. [40], Richmond [41], and Cuaresma et al. [10] that cultivation in short light-path bioreactors is advantageous.

### Engineering strategies for maximal biomass productivity

**Fig. 5:**
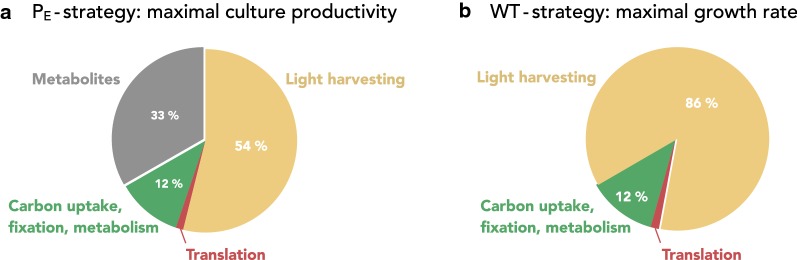
Comparison of the cellular composition for different proteome allocation strategies. **a** $\text {P}_{\mathrm{E}}$-strategy: the protein allocation optimized for maximal culture productivity at a dilution rate of $D=0.026\ \text {h}^{-1}$ with $I_0=440\ \upmu \text {E m}^{-2}\ \text {s}^{-1}$. **b** WT-strategy: the protein allocation is optimized to give rise to a maximal effective growth rate for the respective average light intensity at the same dilution rate and incident light intensity. The $\text {P}_{\mathrm{E}}$-strategy results in a significant reduction of light-harvesting protein complexes (PSU). Instead, intracellular metabolites are accumulated. For simplicity, the cellular composition is shown without (protein and metabolic) quota components (quota components correspond to $\approx 77.2\%$ of cellular dry weight)

Different from phenomenological growth models, the coarse-grained model allows us to investigate the proteome allocation strategies that maximize culture productivity ($\text {P}_{\mathrm{E}}$-strategy)—and to compare the respective differences from the allocation strategy that maximizes growth rate (WT-strategy). Figure 5 shows the optimal proteome allocation for both optimization strategies, performed at a dilution rate of $D=0.026\ \text {h}^{-1}$ for an incident light intensity $I_0 = 440\ \upmu \text {E m}^{-2}\ \text {s}^{-1}$. For simplicity, the figure neglects quota components ($55\%$ of cell mass corresponds to non-protein components, represented by the metabolic compound $c_q$, $50\%$ of protein mass corresponds to quota protein). Cells optimized for culture productivity exhibit a reduced expression of the photosynthetic unit (PSU), and instead accumulate free metabolites. Other proteome components are similar for both optimization strategies.

**Fig. 6:**
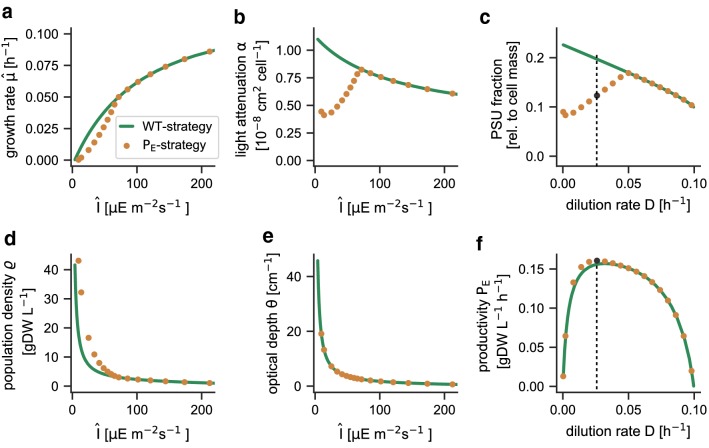
Comparison of cellular and culture properties between the $\text {P}_{\mathrm{E}}$-strategy and WT-strategy. Proteome allocation was optimized for culture productivity for different dilution rates *D* and $I_0=440\ \upmu \text {E m}^{-2}\ \text {s}^{-1}$, and compared to the respective values obtained for the WT-strategy. **a** The effective growth rate as a function of the average light intensity $\hat{I}$. The $\text {P}_{\mathrm{E}}$-strategy results in a reduced effective growth rate at low effective light intensities. **b** The cellular light attenuation coefficient $\alpha$. **c** The fraction of protein PSU (light harvesting and photosynthesis) as a fraction of cell mass. **d** The resulting population density $\varrho$ as a function of the average light intensity. **e** The optical depth $\theta = \alpha \cdot {\varrho }$ of both cultures. **f** The overall volumetric productivity of both optimization strategies as a function of the dilution rate. Despite the significant differences in proteome allocation, the differences in volumetric productivity remain small

Figure 6 provides a more detailed comparison between both optimization strategies for different dilution rates *D*. The results are shown as a function of the resulting average light intensity. Figure 6a shows that strains optimized for culture productivity ($\text {P}_{\mathrm{E}}$-strategy) exhibit a slightly lower effective growth rate $\hat{\mu }$ as a function of the effective average light intensity compared to strains optimized for maximal growth (WT-strategy). This difference is due to different proteome allocation. As shown in Fig. 6b, cells optimized for culture productivity also exhibit a lower light attenuation coefficient $\alpha$. Correspondingly, the culture exhibit a higher population density $\varrho$ at the same effective light intensity (Fig. 6d). The optical depth of both cultures, defined as $\theta = \alpha \cdot {\varrho }$, remains unchanged (Fig. 6e) and the overall light absorption of both cultures is identical.

The difference in proteome allocation as a function of the dilution rate is again shown Fig. 6c: the lower light absorption coefficient of cells optimized for maximal culture productivity results from the fact that these cells express less PSU. The differences in protein allocation between both strains are restricted to low dilution rates (including the dilution rate at which the maximal productivity is attained). For higher dilution rates, both optimization strategies give rise to an identical proteome allocation. The reason for the observed convergence of allocation strategies is that for higher dilution rates *D*, the cells have to allocate increasing resources to ribosomal and metabolic proteins to match the growth rate imposed by the dilution rate.

As shown in Fig. 6f, however, the quantitative differences between the volumetric productivities of both optimization strategies as a function of the dilution rate, are rather small—despite the significant differences in proteome allocation. We note that the absolute quantitative difference in maximal productivity may also be strain-specific and may depend on the parameterization of the model. Figures 5 and 6 show results for an incident light intensity of $I_0=440\ \upmu \text {E m}^{-2}\ \text {s}^{-1}$, all results remain qualitatively identical for other values of $I_0$.

### Sensitivity analysis

**Fig. 7:**
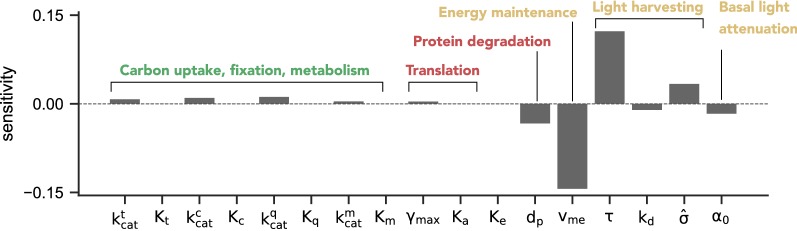
Sensitivity analysis of the maximal productivity $P_E$ with respect to model parameters. Shown is the relative (logarithmic) sensitivity of $P_E$ with respect to variation in kinetic parameters, using an incident light intensity of $I_0=440\ \upmu \text {E m}^{-2}\ \text {s}^{-1}$. The results for other incident light intensities are qualitatively similar. The dilution rate *D* was allowed to vary as part of the optimization problem

To obtain further insights to what extend parameters other than proteome allocation affect the maximal productivity, we performed a sensitivity analysis of the maximal productivity with respect to the model parameters. For details on the estimation see “Methods” section. The results, shown in Fig. 7, indicate that parameters that positively influence growth rate also improve overall productivity. In particular, a larger catalytic activity $\tau$ (catalytic cycles per second) of the PSU increases productivity. Likewise, an increase of the effective cross section $\hat{\sigma }$ per PSU results in an increased productivity, contrary to arguments suggested in the context of antennae truncation. The influence of most other catalytic activities $k_{\mathrm{cat}}$ is modest. From a mechanistic perspective, parameter changes that increase the growth rate at a given average light intensity allow the model to attain the required growth rate (set by the dilution rate *D*) at a lower average light intensity, resulting in a higher culture density and hence a higher productivity. Vice versa, parameters that are expected to negatively affect growth, such as an increased protein degradation constant $d_p$, increased basal maintenance $v_{me}$, and increased photoinhibition constant $k_d$, as well as an increased basal light attenuation $\alpha _0$ all reduce the maximal productivity.

### Engineering strategies for heterologous production

In addition to the production of biomass, cyanobacteria are potential host organisms for the light-driven heterologous synthesis of bioproducts. Metabolic engineering for heterologous production, however, requires optimal expression strategies. To explore the trade-offs between growth and optimal product synthesis, we extend the coarse-grained model with an additional enzyme complex $E_X$ (representing a set of heterologous proteins) that catalyzes the synthesis and excretion of a product of interest. In brief, we introduce a reaction $v_x$, catalyzed by $E_X$, that uses the carbon precursor $c_3$ as substrate and exports a metabolite $m_x$ into the extracellular medium. The compound $m_x$ represents a small molecule of interest, such as lactate [2], ethanol [12], or a volatile product [55], whose heterologous production has been achieved in cyanobacteria. For simplicity, we neglect effects of product inhibition or toxicity [25], but these could be readily incorporated into the definition of $v_x$ if the respective data are available. See “Methods” section for model definitions.

We are interested in the (maximal) volumetric productivity $P_X = v_x \cdot {\varrho }$, defined as the synthesis rate per cell multiplied by the population density. We note that the definition of $P_X$ holds independently on how the product is removed from the medium, i.e., whether the product is removed as part of the output flux ($D_x = D$) or with a separate rate $D_x \ne D$. In either case, the mass-balance equation holds,

14 $$\begin{aligned} 0 = v_x \cdot {\varrho } - D_x \cdot [m_x], \end{aligned}$$

and the concentration of $m_x$ adjusts accordingly.

**Fig. 8:**
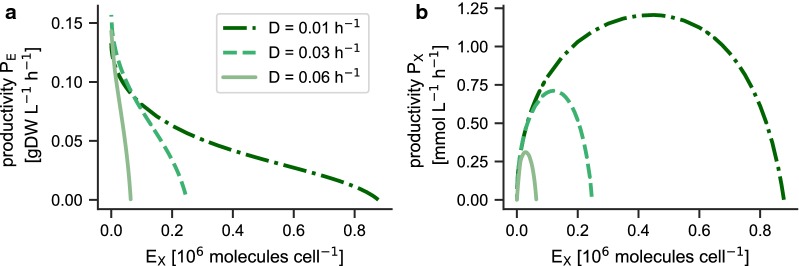
Engineering strategies for heterologous production. The heterologous expression of a protein $E_X$ that catalyses the synthesis and export of a product of interest is enforced. **a** Shown is the volumetric biomass productivity as a function of the expression of the heterologous protein. As expected, increasing expression results in lower biomass productivity (‘the protein burden’). **b** The resulting productivity $P_X$ of the compound of interest. $P_X$ exhibits a maximum as a function of heterologous expression. In both plots $I_0= 440\ \upmu \text {E m}^{-2}\ \text {s}^{-1}$

We first consider the trade-off between expression of the heterologous protein $E_X$, biomass productivity (the ‘protein burden’), and $P_X$ respectively. To this end, we force the heterologous expression of the protein $E_X$ within the WT-strain by introducing a lower bound on its concentration (in molecules per cell) as an additional constraint into the optimization objective, and subsequently use the WT-strategy to maximize the effective growth rate. The optimization establishes a ’best case’ scenario for growth under the constraint of heterologous expression. Fig. 8a shows the resulting trade-off between biomass productivity and expression for three different dilution rates *D*. As expected, biomass productivity decreases with increasing heterologous expression and there is a maximal expression after which the biomass productivity ceases: the remaining proteome resources are not sufficient to ensure a specific growth rate that matches the dilution rate *D* and the culture is washed out. Figure 8b shows the resulting productivity $P_X$ as a function of protein expression. The productivity $P_X$ exhibits a maximum for a specific expression of $E_X$ that depends on the dilution rate *D*.

**Fig. 9:**
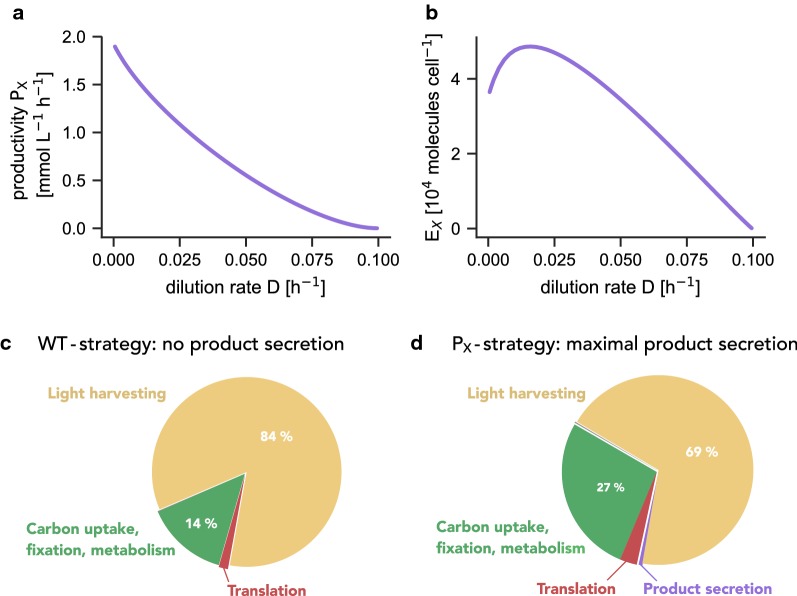
Maximal productivity of heterologous production. We consider a hypothetical strain whose protein allocation maximizes the productivity $P_X$ for different dilution rates *D*. Results are shown for an incident light intensity $I_0 = 440\ \upmu \text {E m}^{-2}\ s^{-1}$. **a** The maximal productivity $P_X$ decreases with increasing dilution rate *D*. **b** The optimal expression of the heterologous protein as a function of dilution rate. **c** The cellular composition for the WT-strategy for $I_0 = 440\ \upmu \text {E m}^{-2}\ \text {s}^{-1}$ and a dilution rate $D=0.03\ \text {h}^{-1}$. **d** Optimal cellular composition under the $\text {P}_{\mathrm{X}}$-strategy for an identical incident light intensity and dilution rate

Beyond enforced expression, we are interested in the predicted proteome allocation of a hypothetical $\text {P}_{\mathrm{X}}$-strain that maximizes the productivity $P_X$—and how this predicted proteome differs from the proteome of a WT-strain. To this end, $P_X$ is maximized for different dilution rates *D* as a function of protein expression, i.e., by varying the fraction $\beta _j$ of ribosomes that translate a specific protein $P_j$. The results are summarized in Fig. 9. Figure 9a shows the maximal productivity $P_X$ of the optimized $\text {P}_{\mathrm{X}}$-strain as a function of dilution rate *D*. The maximal productivity $P_X$ decreases with increasing dilution rates: for heterologous production cyanobacteria act as catalysts and the maximally productive state of the culture is attained when growth (almost) ceases (i.e., the reactor has a low dilution rate) and all cellular resources are directed to carbon assimilation and product synthesis. This finding holds independently of the removal rate of the product. In practice, however, product inhibition and possible toxicity of the accumulated dissolved products will either prohibit very low dilution rates or necessitate fast removal of the product. Fig. 9b shows the optimal heterologous expression necessary for a maximal productivity $P_X$. Figures 9c, d compare the cellular composition of cells optimized for maximal growth (WT-strategy) and maximal productivity $P_X$ ($\text {P}_{\mathrm{X}}$-strategy) at the same dilution rate *D*. In the latter case, protein complexes associated to light harvesting and photosynthesis (PSU) are again reduced, whereas protein complexes associated to carbon uptake and assimilation are increased.

## Discussion

In this study, our aim was to provide insights into the limits of phototrophic cultivation of microorganisms in a light-limited chemostat. To this end, we built upon an established theory of growth in a light-limited chemostat, as developed by Huisman et al. [23] and others [18, 30, 31]. Previous analyses, however, primarily relied on phenomenological growth models, such as the Monod or Haldane-type equation. In contrast, our starting point was a mechanistic model of cyanobacterial growth that connects intracellular resource allocation with physiological properties and growth. The model was previously parameterized using data obtained from an (optically thin) turbidostat culture of the cyanobacterium *Synechocystis* sp. PCC 6803, and was subject to a detailed analysis with respect to the predicted physiological properties as a function of growth rate and light intensity [54]. Our premise was therefore that the model represents a reasonable description of cyanobacterial growth at different light intensities. Our aim was to extrapolate the results obtained from the coarse-grained single-cell model to dense cultures that give rise to strong light gradients due to self-shading—motivated by the hypothesis that growth in an optically dense culture imposes different trade-offs on resource allocation. Following previous works [23], and the experimental setup used by Zavřel et al. [54], we only considered monochromatic light (the computational approach, however, can be straightforwardly extended to different light spectra).

Our first step was to integrate the coarse-grained growth model into a model of the light-limited chemostat. The rate equations as a function of the light gradient were solved analytically, resulting in a description of phototrophic growth that only depends on the average light intensity within the chemostat. Such a description was previously utilized by several authors, for example Du et al. [13] and Clark et al. [7], as a reasonable approximation. Our results, however, provide a more stringent justification for this approximation: it emerges as a direct consequence of the model definitions. A crucial prerequisite for this fact is that photodamage is assumed to happen at all light intensities and that the rate constant of photodamage (the degradation of the PSU protein complex that represents the degradation of the D1 protein) is directly proportional to light intensity. There is indeed significant experimental evidence for this assertion: the finding that the rate constant of photodamage is directly proportional to light intensity has been confirmed several times in various organisms [6, 32, 44–46]. It has already been highlighted [44, 45] that the first-order behavior of photoinhibition is not a trivial result, and runs contrary to the belief that photoinhibitory damage does not occur under low light. We note that the first-order dependence is an empirical finding that is independent of details of the model implementation.

Within our computational framework, the first-order dependence allows us to solve the model analytically. The solution has strong implications for phototrophic cultivation and the design of photobioreactors. Firstly, these results provide a stringent justification for the photonfluxostat [13] as a suitable tool for quantitative growth experiments. In particular, the average light intensity, as defined in Eq. (11), can be readily estimated experimentally using the incident and transmitted light intensities, $I_0$ and $I(z_m)$ respectively, and therefore may provide direct feedback to a controller. The definition provided in Eq. (11) also provides a more accurate description than the approximations previously used in phenomenological growth models [7].

Secondly, and more importantly, the dependence on the average light intensity implies that the culture density itself provides an effective mechanism of light dilution. If photodamage is directly proportional to light, then the average rate of photodamage equals the photodamage rate at the average light intensity—with the latter being determined by the culture density. For a rapidly mixed culture, our model therefore predicts maximally efficient growth for high densities at very high light intensities. Within the model, higher light intensity will always result in denser cultures with no obvious upper bound imposed by the model itself (a fact that is different to the analysis of Martínez et al. [31] where the maximal productivity has an upper bound independent of the light intensity). In practice, however, we expect that at high culture densities the supply of other nutrients, in particular inorganic carbon, becomes limiting—resulting in a *de facto* upper bound on the feasible cell density that is outside the scope of the current model.

Our model predictions can be compared to growth data reported in the literature. Results obtained from conventional cultivation typically report significantly lower cell densities compared to the values suggested here. See, for example, Straka and Rittmann [42] for typical values for *Synechocystis* sp. PCC 6803 cultured in conventional BG-11 medium (the comparison with our prediction is shown in Additional file 1: Figure S3). However, several recent works have shown that conventional BG-11 media is not suitable for high density cultivation and alternatives are required [3, 29, 48, 52]. Previous works have shown that cultivation of *Synechocystis* sp. PCC 6803 is feasible at cell densities in excess of $20\ \text {gDW L}^{-1}$ and light intensities in excess of $1000\ \upmu \text {E m}^{-2}\ \text {s}^{-1}$ with no apparent detrimental effects due to photoinhibition [3, 29]. Similar results were recently reported for other cyanobacterial strains [52]. The predictions of our model in favor of cultivation at very high light intensities in shallow rapidly mixed cultures are also confirmed by the experiments of Qiang et al. [40] using *Spirulina platensis*. Therein a linear relationship was observed between the output rate (in $\text {gDW L}^{-1}\ \text {h}^{-1}$) and the incident light intensity, up to a photon flux of $2500\ \upmu \text {E m}^{-2}\ \text {s}^{-1}$, with areal productivities similar to the values computed here. Taken together, these results strongly support the previous arguments of Richmond [41] for cultivation at high light intensities in shallow rapidly mixed cultures with short light-paths for maximal phototrophic productivity.

Beyond the argument for dense cultures, the model recapitulates many of the results previously obtained for the light-limited chemostat using phenomenological growth models. In particular, we recover the observed bistability for incident light intensities that give rise to photoinhibition. While we were primarily interested in the steady-state properties, bistability has complex implications for the startup and dynamics of a culture [18, 30, 31], for example a threshold in the (initial) population density below which the culture will wash out.

In our analysis, we were further interested in the optimal proteome allocation for phototrophic production. As a benchmark for comparison, we assume that wild-type cells adjust their proteome composition such that they achieve the maximal growth rate at the respective average light intensity (WT-strategy). As shown in Fig. 6, the WT-strategy is (evolutionary) stable with respect to alternative proteome allocation strategies. Our results show that the composition of (hypothetical) strains optimized for maximal biomass productivity differs significantly from the composition of cells using the WT-strategy. Maximally productive strains exhibit a significantly reduced expression of protein complexes associated with light harvesting and photosynthesis (protein complex PSU). This reduction is reminiscent of antennae truncation strategies [33]: the WT-strategy maximized growth rate at the expense of culture efficiency. If the light absorption per cell is reduced, the population density of the culture increases, and hence the productivity increases. Interestingly, the reduction in light-harvesting proteins, however, does not result in an increase of other protein fractions, but rather in the accumulation of free metabolites (Fig. 5). This unintuitive result arises because the cellular growth rate within a chemostat is determined by the dilution rate. Hence the required (minimal) capacity for metabolic and ribosomal proteins is fixed. To fulfill the density constraints imposed within the model, the cell therefore accumulates free metabolites.

We further observed that, despite the significant differences in cellular composition, the quantitative differences in productivity between cells optimized for biomass productivity and the WT-strategy are rather small. This difference, however, may be strain-dependent and coarse-grained growth models parameterized for other data or strains data might exhibit larger differences. The small (and possible strain-dependent) difference in the overall productivity might also explain the mixed success reported for antenna truncation [28]. As shown in Fig. 7, a simple reduction in the effective cross section per PSU does not result in an enhanced productivity.

Noteworthy, the maximal culture productivity is typically attained at dilution rates well below the maximal growth rate of cells. This finding has implications for the current quest to identify the fastest growing cyanobacterium [47, 53]—with grow rates typically measured in optically thin cultures under optimal conditions. While growth rate still remains an important parameter, our results show that culture productivity is determined by a combination of factors, including maximal culture density, dilution rate, and incident light intensity. We envision that the computational framework presented here may be further developed into an automated “design-build-test-learn” pipeline for microbial design strategies that allows to extrapolate the expected culture productivity of strains based on a defined set of screening experiments.

## Conclusion

The results obtained from our computational model have strong implications for phototrophic cultivation and the design of photobioreactors. The (experimentally well supported) fact that the rate of photodamage is directly dependent on light intensity implies that phototrophic growth in the light-limited chemostat can be efficiently described using the concept of an average light intensity. Furthermore, the first-order dependency of photodamage implies that the culture density itself provides sufficient light dilution—given that the cells are rapidly mixed and other nutrients are available in non-limiting concentrations. We have previously shown that suitable cultivation setups are indeed possible by combining short light-paths (up to 1 cm) with high light intensities ($>1000\ \upmu \text {E m}^{-2}\ \text {s}^{-1}$), turbulent mixing and sufficient supply of inorganic nutrients [3, 29]. As already emphasized by Richmond [41], such results rekindle the hope that growing algae and cyanobacteria in ultra high densities may boost economic viability of phototrophic cultivation. The early experimental results of Qiang et al. [40] are recovered here and put in the context of a thorough computational framework that builds upon recent insights in the cellular economy of phototrophic growth [54].

The computational framework presented here provides a further step to guide phototrophic cultivation and the development of phototrophic cell factories. While our study was limited to steady-state conditions, further work may assess the dynamics of cellular proteome allocation and the resulting population dynamics. Furthermore, in future work, the model may be extended to incorporate further molecular details, in particular with respect to the photosynthetic light reaction, cycling of inorganic carbon, photorespiration, storage metabolism, oxygen accumulation, as well as the potential effects of product toxicity and inhibition. We conjecture that our approach will prove useful in understanding the limitations of phototrophic culture productivity and allows us to further optimize culture conditions and cellular composition.

## Methods

### A model of phototrophic growth

We use the previously described model of Faizi et al. [15] with minor modifications. The model is implemented as an ordinary differential equation (ODE), all parameters are summarized in Additional file 1: Table S1. The model describes phototrophic growth of a cyanobacterial cell and consists of 7 coarse-grained protein complexes that catalyze cellular reactions, as well as 5 intracellular metabolites. Cellular processes require a cellular energy unit *e* that combines ATP and NADPH and is produced by the photosynthetic light reactions.

The metabolic reactions and the stoichiometry of the translation reaction are summarized in Table 2. All metabolic reactions $v_{met}$ are assumed to follow irreversible Michaelis–Menten kinetics,

15 $$\begin{aligned} v_{met} = [E] \cdot k_{cat} \cdot \frac{[m]}{K_m+[m]} \cdot \frac{[e]}{K_e+[e]}, \end{aligned}$$

where [*E*] denotes the concentration of the respective catalyzing protein complex, [*m*] the concentration of the substrate, $K_m$ and $k_{\mathrm{cat}}$ the respective kinetic constants. The Michaelis-Menten $K_e$ constant with respect to the energy unit *e* is assumed to be equal for all reactions.

**Table 2:** Summary of metabolic and translation reactions

Protein	Reaction	Stoichiometry	Description
$E_T$	$v_t$	$c_i^x + e \rightarrow c_i$	Inorganic carbon uptake
$E_C$	$v_c$	$3\cdot c_i + 23 \cdot e \rightarrow c_3$	Carbon assimilation
$E_M$	$v_m$	$2\cdot c_3 + 22 \cdot e \rightarrow aa$	Metabolism
$E_Q$	$v_q$	$c_3 + e \rightarrow c_q$	Synthesis of quota compounds
*R*	$\gamma _j$	$n_j\cdot aa + 3 \cdot n_j \cdot e \rightarrow P_j$	Translation by ribosomes

The protein complex $E_T$ imports extracellular inorganic carbon and represents import and carbon concentrating mechanisms. The protein complex $E_C$ catalyzes assimilation of inorganic carbon into the carbon precursor $c_3$ (Calvin–Benson cycle). The protein complex $E_M$ catalyzes the synthesis of amino acids (central metabolism), whereas the protein complex $E_Q$ catalyzes the synthesis of other metabolic compounds $c_q$. The remaining two protein complexes are the photosynthetic unit (PSU) and a (non-functional) quota protein compound $P_Q$

For each protein complex $P_j$, the translation rate $\gamma _j$ depends on the length $n_j$ of the protein (in units of amino acids, aa) and the fraction $\beta _j \cdot [R]$ of ribosomes that translate protein $P_j$ (with $\sum \beta _j \le 1$),

16 $$\begin{aligned} \gamma _j =\beta _j \cdot [R] \cdot \frac{\gamma _{max}}{n_j} \cdot \frac{[aa]}{K_a+[aa]} \cdot \frac{[e]}{K_e+[e]}, \end{aligned}$$

where $\gamma _{max}$ denotes the maximal catalytic rate of the ribosome. The parameters $\beta _j$ determine cellular proteome allocation.

All intracellular compounds are subject to dilution by growth. The dynamics of the protein complexes are further determined by their respective translation rate $\gamma _j$ and an additional degradation term $d_p$,

17 $$\begin{aligned} \frac{d[P_j]}{dt} = \gamma _{j} - (\mu + d_p) \cdot [P_j]. \end{aligned}$$

The equation holds for all protein complexes, except the PSU. The photosynthetic unit *PSU* additionally includes fast transitions between an activated $PSU^{*}$ and resting state $PSU^o$ with ODEs

18 $$\begin{aligned} \frac{d[PSU^o]}{dt} = \gamma _{PSU} + v_2 - \frac{v_1}{m_{hv}} - (\mu + d_p) \cdot [PSU^o], \end{aligned}$$

and

19 $$\begin{aligned} \frac{d[PSU^{*}]}{dt} = \frac{v_1}{m_{hv}} - v_2 - v_i - (\mu + d_p) \cdot [PSU^{*}]. \end{aligned}$$

The activated state $PSU^{*}$ may undergo light-dependent degradation with a rate $v_i$ (photodamage). The respective rate equations are

20 $$\begin{aligned} v_1= &\, {} \hat{\sigma } \cdot I \cdot [PSU^o], \end{aligned}$$

21 $$\begin{aligned} v_2= &\, {} \tau \cdot [PSU^{*}], \end{aligned}$$

22 $$\begin{aligned} v_i= &\, {} k_d \cdot \hat{\sigma } \cdot I \cdot [PSU^{*}], \end{aligned}$$

where $\tau$ denotes the catalytic turnover of the PSU, $\hat{\sigma }$ the effective cross section for light absorption, and $k_d$ a rate constant for photodamage. These equations correspond to a three-state model of photosynthesis [14, 50] where degradation and recovery of PSU involves the constituent amino acids. The first-order light dependency of the reactions, in particular for photoinhibition, is strongly supported by data [6, 44]. We emphasize that this fact does not imply that the growth curve or the oxygen evolution rate exhibit first-order dependencies—both are outcomes of a trade-off between different constraints and objectives (see Fig. 1c). The overall equation for photosynthesis converts $m_{hv} =8$ photons into 8 energy units *e* (representing a total of 5 ATP and 2 NADPH, the latter are weighted as 1.5 *e* each) and one $\text {O}_{2}$. The model consists of 13 ODEs, an objective function (see below), and additional constraints to ensure synthesis of quota compounds (a quota protein $P_Q$ and the metabolic compound $c_q$). External parameters are the concentration of extracellular inorganic carbon and the light intensity $I_0$. The former is assumed to be constant and saturating with respect to the $K_t$ of the transporter reaction.

### Solving the light gradient

To obtain an expression of the effective growth rate $\hat{\mu }$ in the light-limited chemostat, we require a solution of Eq. (10) and integrate the rate equations over the mixing depth of the photobioreactor. Following [23], we use a substitution of variables with

23 $$\begin{aligned} \frac{dI}{dz} = - (\alpha \cdot \varrho + K_{bg} )\cdot I, \end{aligned}$$

to replace the integral over the mixing depth with an integral over the light intensities,

24 $$\begin{aligned} \hat{\mathbf{v}} {({\varvec{\beta }}, \mathbf{x}, \hat{I})} =\frac{1}{z_m \cdot (\alpha \cdot \varrho + K_{bg})} \cdot \int _{{I(z_m)}}^{I_0} \frac{\mathbf{v} {({\varvec{\beta }}, \mathbf{x}, I)}}{I}\ \ dI, \end{aligned}$$

and note that

25 $$\begin{aligned} \ln (I_0) - \ln ({I(z_m)}) = z_m \cdot (\alpha \cdot \varrho + K_{bg}). \end{aligned}$$

We distinguish between light-independent reactions and reactions affected by light. The former remain unchanged, whereas the latter only consist of reactions that exhibit a first-order dependency on the light intensity ($v_1$ and $v_i$). Consequently, the solution for each reaction rate replaces the depth-dependent light intensity with the average light intensity $\hat{I}$, defined in Eq. (11), in the chemostat. The derivation is based on the assumption that no further variables depend on the (momentary) position in the chemostat, i.e., the culture is rapidly mixed.

### Sensitivity analysis

The sensitivity analysis is performed to quantify the influence of model parameters on culture productivity. The relative sensitivity $\epsilon _i$ of the culture productivity $P_E$ with respect to a given model parameter $p_i$ is defined as

26 $$\begin{aligned} \epsilon _i = \frac{\Delta P_E}{P_E} \cdot \frac{p_i}{\Delta p_i}, \end{aligned}$$

and approximated by a small variation ($\pm 0.1\%$) of each parameter $p_i$. A relative sensitivity of $\epsilon _i =1$ indicates a linear dependency of the culture productivity on the respective parameter $p_i$.

### Heterologous expression

The coarse-grained model is modular and allows for the addition of further enzymes of interest. We extend the model to include the synthesis and export of a desired product $m_x$. For each enzyme complex $E_X$ the following parameters have to be defined: enzyme length $n_x$, its turnover rate $k_{cat}^x$ and other kinetic parameters (here the half-saturation constant $K_x$ with respect to its substrate). In this case, we assume irreversible Michaelis–Menten kinetics,

27 $$\begin{aligned} v_x = {[E_X]} \cdot k_{cat}^x \cdot \frac{{[c_3]}}{K_x+{[c_3]}} \cdot \frac{{[e]}}{K_e+{[e]}}, \end{aligned}$$

the new reaction has to be added to the ODEs for the respective substrates (here $c_3$ and *e*) and product (here $m_x$). It is straightforward to also include, for example, a term for product inhibition into the equation provided the respective parameters are known. The model is augmented by two additional ODEs,

28 $$\begin{aligned} \frac{d[m_x]}{dt} = v_x \cdot \varrho - D \cdot [m_x]\ , \end{aligned}$$

and

29 $$\begin{aligned} \frac{d[E_X]}{dt} = \gamma _{E_X} - (\mu + d_p) \cdot [E_X]\ . \end{aligned}$$

with a translation rate $\gamma _{E_X}$. The protein complex $E_X$ competes with other proteins for ribosomal capacity and is included into the definition of the cell density.

### Model parametrization

The model parameters are taken from the previously published models [15, 54], only the turnover rate of the photosynthetic unit $\tau$, its effective absorption cross section $\hat{\sigma }$ and the photodamage constant $k_d$ are refitted in this study. We first performed a rough estimate for the photosynthetic turnover rate $\tau = 500\ \text {s}^{-1}$. Additional file 1: Figure S1 shows how different turnover rates effect the growth rate for arbitrarily chosen photodamage constant $k_d$ and absorption cross section $\hat{\sigma }$. Fitting of the remaining parameters $k_d$ and $\hat{\sigma }$ was performed as described in Faizi et al. [15] for a predefined set of values $k_d = \{ 10^{-7}, 1.1 \cdot 10^{-7} ..., 4.9 \cdot 10^{-7}, 5 \cdot 10^{-7} \}$ and $\hat{\sigma } = \{5, 10, ..., 25, 30\}$. The best fit was obtained with $k_d = 2.7\cdot 10^{-7}$ and $\hat{\sigma } = 15\ \text {nm}^{2}\ \text {PSU}^{-1}$.

All parameters are listed in Additional file 1: Table S1. Unless otherwise noted, the average length of a protein is 300 amino acids, the average turnover rate is $k_{cat} = 20\ \text {s}^{-1}$ and the average half-saturation constant is set to $10^4$ molecules per cell. In addition, we assume that the amount of photons required to activate one PSU is $m_{hv} = 8$ photons. With respect to the measurements from Zavřel et al. [54], we set the concentration of quota compounds to $10^{11}$ molecules of carbon per cell. The concentration of quota compounds represent the amount of carbon molecules contained in the dry weight per cell without proteins.

To parameterize the chemostat model we determined the light attenuation through the culture vessel filled only with medium. For this purpose, we fitted Eq. 3 to the light profile data in Additional file 1: Figure S2, for a vessel depth of $z_m = 2.4\ \text {cm}$ without microorganisms ($\alpha \cdot \varrho = 0$) and obtain the background turbidity for the culture medium of $K_{bg} = 0.06\ \text {cm}^{-1}$. The species-specific basal light attenuation coefficient is set to $\alpha _0 = 0.01\ \upmu \text {m}^{2}$ per cell, which is approximately one order of magnitude smaller than the varying light attenuation coefficient determined by the total photosynthetic unit amount and the absorption cross section at high light conditions.

The units for culture density are gram dry weight per liter (gDW/L). We note that the original measurements and parametrization of Zavřel et al. [54] was in cells per liter, and the conversion into gDW is subject to considerable variance, owing to the experimental difficulties in the accurate estimation of dry weight. Replicate measurements reported in Zavřel et al. [54] vary from $0.53 \cdot 10^{-11}$ gDW/cell to $1.13 \cdot 10^{-11}$ gDW/cell. In this work, we assume a conversion factor of $10^{-11}$ gDW/cell, for visual clarity error bars are omitted in all plots (but see Additional file 1: Figure S3 for an example of error ranges). We emphasize that our aim is not a precise prediction of (the numerical value of) a specific productivity, but rather to investigate the dependence of the (maximal) productivity on culture parameters—these results are independent of the conversion factor.

### Model implementation

The model is implemented as an optimization problem to obtain the optimal proteome allocation for a specific environmental condition, characterized by the incident light intensity $I_0$, external inorganic carbon concentration $c_i^x$, mixing depth $z_m$ and dilution rate *D*. The variable parameters in our optimization problem are the population density $\varrho$ and the ribosomal fractions $\beta _j$. For the objective function of our optimization problem we first assume that the cell optimizes the internal composition such that the growth rate is maximal for the specific external condition (WT-strategy). In addition, we define two further objective functions. The second objective function maximizes the product of the dilution rate and population density ($P_E = D \cdot \varrho$) to determine the optimal proteome allocation that maximizes the volumetric biomass productivity of the culture. The third objective function maximizes the productivity of a desired product $m_x$ ($P_X = v_x \cdot \varrho$).

The optimization problem is implemented with the APMonitor Optimization Suite [21] and solved using the IPOPT (Interior Point Optimizer) method. The model is written in the APMonitor modeling language and provided on https://github.com/marjanfaizi/photoautotrophic-growth (in the folder ‘Faizi2019’) together with a Python script (entitled optimization.py) to run the simulations.

## Supplementary information

**Additional file 1: Figure S1.** Parameter fitting. **Figure S2.** Light profile of photobioreactor filled with medium only. **Figure S3.** Prediction errors and comparison of in silico results with experimental data using *Synechocystis* sp. PCC 6803. **Table S1.** Model parameters taken from Faizi et al. [15]^●^, Zavrel et al. [54]^○^ or estimated here.

## Data Availability

All data generated or analysed during this study are included in this published article [and its additional information files]. The code used in the simulations is available at https://github.com/marjanfaizi/photoautotrophic-growth.

## References

[1] Andersson B, Shen C, Cantrell M, Dandy DS, Peers G (2019). The fluctuating cell-specific light environment and its effects on cyanobacterial physiology. Plant Physiol..

[2] Angermayr SA, Paszota M, Hellingwerf KJ (2012). Engineering a cyanobacterial cell factory for production of lactic acid. Appl Environ Microbiol.

[3] Bähr L, Wüstenberg A, Ehwald R (2016). Two-tier vessel for photoautotrophic high-density cultures. J Appl Phycol.

[4] Bernard O (2011). Hurdles and challenges for modelling and control of microalgae for $$\text{CO}_{2}$$ mitigation and biofuel production. J Process Control.

[5] Burnap RL (2015). Systems and photosystems: cellular limits of autotrophic productivity in cyanobacteria. Front Bioeng Biotechnol.

[6] Campbell DA, Tyystjärvi E (2012). Parameterization of photosystem II photoinactivation and repair. Biochim et Biophys Acta (BBA) Bioenerg.

[7] Clark RL, McGinley LL, Purdy HM, Korosh TC, Reed JL, Root TW, Pfleger BF (2018). Light-optimized growth of cyanobacterial cultures: growth phases and productivity of biomass and secreted molecules in light-limited batch growth. Metab Eng.

[8] Cordara A, Re A, Pagliano C, Van Alphen P, Pirone R, Saracco G, Branco Dos Santos F, Hellingwerf K, Vasile N (2018). Analysis of the light intensity dependence of the growth of *Synechocystis* and of the light distribution in a photobioreactor energized by 635 nm light. PeerJ.

[9] Cornet J-F, Dussap C-G (2009). A Simple and reliable formula for assessment of maximum volumetric productivities in photobioreactors. Biotechnol Prog.

[10] Cuaresma M, Janssen M, Vílchez C, Wijffels RH (2009). Productivity of Chlorella sorokiniana in a short light-path (SLP) panel photobioreactor under high irradiance. Biotechnol Bioeng.

[11] de Jong H, Casagranda S, Giordano N, Cinquemani E, Ropers D, Geiselmann J, Gouze JL (2017). Mathematical modelling of microbes: metabolism, gene expression and growth. J R Soc Interface.

[12] Dexter J, Fu P (2009). Metabolic engineering of cyanobacteria for ethanol production. Energy Environ Sci.

[13] Du W, Jongbloets JA, Hernández HP, Bruggeman FJ, Hellingwerf KJ, dos Santos FB (2016). Photonfluxostat: a method for light-limited batch cultivation of cyanobacteria at different, yet constant, growth rates. Algal Res.

[14] Eilers P, Peeters J (1988). A model for the relationship between light intensity and the rate of photosynthesis in phytoplankton. Ecol Model.

[15] Faizi M, Zavřel T, Loureiro C, Červený J, Steuer R (2018). A model of optimal protein allocation during phototrophic growth. BioSystems.

[16] Fuente Herraiz D, Keller J, Conejero A, Rögner M, Rexroth S, Urchueguia J (2017). Light distribution and spectral composition within cultures of micro-algae: quantitative modelling of the light field in photobioreactors. Algal Res.

[17] Gao X, Sun T, Pei G, Chen L, Zhang W (2016). Cyanobacterial chassis engineering for enhancing production of biofuels and chemicals. Appl Microbiol Biotechnol.

[18] Gerla DJ, Mooij WM, Huisman J (2011). Photoinhibition and the assembly of light-limited phytoplankton communities. Antonie van Leeuwenhoek.

[19] Han B-P (2002). A mechanistic model of algal photoinhibition induced by photodamage to photosystem-II. J Theor Biol.

[20] He L, Wu SG, Wan N, Reding AC, Tang YJ (2015). Simulating cyanobacterial phenotypes by integrating flux balance analysis, kinetics, and a light distribution function. Microb Cell Fact.

[21] Hedengren JD, Shishavan RA, Powell KM, Edgar TF (2014). Nonlinear modeling, estimation and predictive control in APMonitor. Comput Chem Eng.

[22] Huang Q, Jiang F, Wang L, Yang C (2017). Design of photobioreactors for mass cultivation of photosynthetic organisms. Engineering.

[23] Huisman J, Matthijs HC, Visser PM, Balke H, Sigon CA, Passarge J, Weissing FJ, Mur LR (2002). Principles of the light-limited chemostat: theory and ecological applications. Oikos.

[24] Jahn M, Vialas V, Karlsen J, Maddalo G, Edfors F, Forsstrom B, Uhlen M, Kall L, Hudson EP (2018). Growth of Cyanobacteria is constrained by the abundance of light and carbon assimilation proteins. Cell Rep.

[25] Kamarainen J, Knoop H, Stanford N, Guerrero F, Akhtar M, Aro E-M, Steuer R, Jones P (2012). Physiological tolerance and stoichiometric potential of cyanobacteria for hydrocarbon fuel production. J Biotechnol.

[26] Kehr J-C, Gatte Picchi D, Dittmann (2011). Natural product biosyntheses in cyanobacteria: a treasure trove of unique enzymes. Beilstein J Org Chem.

[27] Khana MI, Shin JH, Kim JD (2018). The promising future of microalgae: current status, challenges, and optimization of a sustainable and renewable industry for biofuels, feed, and other products. Microb Cell Fact.

[28] Lea-Smith DJ, Bombelli P, Dennis JS, Scott SA, Smith AG, Howe CJ (2014). Phycobilisome-deficient strains of *Synechocystis* sp. PCC 6803 have reduced size and require carbon-limiting conditions to exhibit enhanced productivity. Plant Physiol.

[29] Lippi L, Bähr L, Wüstenberg A, Wilde A, Steuer R (2018). Exploring the potential of high-density cultivation of cyanobacteria for the production of cyanophycin. Algal Res.

[30] Martínez C, Bernard O, Mairet F (2018). Maximizing microalgae productivity in a light-limited chemostat. IFAC-PapersOnLine.

[31] Martínez C, Mairet F, Bernard O (2018). Theory of turbid microalgae cultures. J Theor Biol.

[32] Melis A (1999). Photosystem-II damage and repair cycle in chloroplasts: what modulates the rate of photodamage in vivo?. Trends Plant Sci.

[33] Melis A (2009). Solar energy conversion efficiencies in photosynthesis: minimizing the chlorophyll antennae to maximize efficiency. Plant Sci.

[34] Molenaar D, van Berlo R, de Ridder D, Teusink B (2009). Shifts in growth strategies reflect tradeoffs in cellular economics. Mol Syst Biol.

[35] Monod J (1950). La technique de culture continue. Théorie et applications. Annales de l’Institut Pasteur.

[36] Mynderse JS, Moore RE, Kashiwagi M, Norton TR (1977). Antileukemia activity in the osillatoriaceae: isolation of debromoaplysiatoxin from Lyngbya. Science.

[37] Novick A, Szilard L (1950). Description of the chemostat. Science.

[38] Papacek S, Jablonsky J, Petera K (2018). Advanced integration of fluid dynamics and photosynthetic reaction kinetics for microalgae culture systems. BMC Syst Biol.

[39] Pirt SJ (1986). The thermodynamic efficiency (quantum demand) and dynamics of photosynthetic growth. N Phytol.

[40] Qiang H, Zarmi Y, Richmond A (1998). Combined effects of light intensity, light-path and culture density on output rate of spirulina platensis (cyanobacteria). Eur J Phycol.

[41] Richmond A (1996). Efficient utilization of high irradiance for production of photoautotropic cell mass: a survey. J Appl Phycol.

[42] Straka L, Rittmann BE (2018). Effect of culture density on biomass production and light utilization efficiency of *Synechocystis* sp. PCC 6803. Biotechnol Bioeng.

[43] Touloupakis E, Cicchi B, Torzillo G (2015). A bioenergetic assessment of photosynthetic growth of *Synechocystis* sp. PCC 6803 in continuous cultures. Biotechnol Biofuels.

[44] Tyystjärvi E, Aro EM (1996). The rate constant of photoinhibition, measured in lincomycin-treated leaves, is directly proportional to light intensity. Proc Natl Acad Sci USA.

[45] Tyystjärvi E, Mäenpää P, Aro EM (1994). Mathematical modelling of photoinhibition and Photosystem II repair cycle. I. Photoinhibition and D1 protein degradation in vitro and in the absence of chloroplast protein synthesis in vivo. Photosyn Res.

[46] Tyystjärvi E (2008). Photoinhibition of Photosystem II and photodamage of the oxygen evolving manganese cluster. Coord Chem Rev.

[47] Ungerer J, Wendt KE, Hendry JI, Maranas CD, Pakrasi HB (2018). Comparative genomics reveals the molecular determinants of rapid growth of the cyanobacterium *Synechococcus elongatus* UTEX 2973. Proc Natl Acad Sci USA.

[48] van Alphen P, Abedini Najafabadi H, Branco dos Santos F, Hellingwerf KJ (2018). Increasing the photoautotrophic growth rate of *Synechocystis* sp. pcc 6803 by identifying the limitations of its cultivation. Biotechnol J.

[49] Weiße AY, Oyarzún DA, Danos V, Swain PS (2015). Mechanistic links between cellular trade-offs, gene expression, and growth. Proc Natl Acad Sci USA.

[50] Westermark S, Steuer R (2016). Toward multiscale models of cyanobacterial growth: a modular approach. Front Bioeng Biotechnol.

[51] Wijffels RH, Barbosan MJ (2010). An outlook on microalgal biofuels. Science.

[52] odarczyk A, Selão TT, Norling B, Nixon PJ (2019). Unprecedented biomass and fatty acid production by the newly discovered cyanobacterium *Synechococcus* sp. PCC 11901. bioRxiv.

[53] Yu J, Liberton M, Cliften PF, Head RD, Jacobs JM, Smith RD, Koppenaal DW, Brand JJ, Pakrasi HB (2015). *Synechococcus elongatus* UTEX 2973, a fast growing cyanobacterial chassis for biosynthesis using light and $$\text{ CO }_{2}$$. Sci Rep.

[54] Zavřel T, Faizi M, Loureiro C, Poschmann G, Stühler K, Sinetova M, Zorina A, Steuer R, Červený J (2019). Quantitative insights into the cyanobacterial cell economy. eLife.

[55] Zavřel T, Knoop H, Steuer R, Jones P, Červený J, Trtilek M (2016). A quantitative evaluation of ethylene production in the recombinant cyanobacterium *Synechocystis* sp. PCC 6803 harboring the ethylene-forming enzyme by membrane inlet mass spectrometry. Bioresour Technol.

[56] Zavřel T, Červený J, Knoop H, Steuer R (2016). Optimizing cyanobacterial product synthesis: meeting the challenges. Bioengineered.

[57] Zijffers J-WF, Schippers KJ, Zheng K, Janssen M, Tramper J, Wijffels RH (2010). Maximum photosynthetic yield of green microalgae in photobioreactors. Mar Biotechnol.

