# Supplementary material for: Optimal proteome allocation strategies for phototrophic growth in a light-limited chemostat
Source: Microb Cell Fact. 2019 Oct 10;18:165. doi: 10.1186/s12934-019-1209-7 (PMC6785936; doi:10.1186/s12934-019-1209-7)
Supplement: Supplementary file 1 — Additional file 1: Figure S1. Parameter fitting. Figure S2. Light profile of photobioreactor filled with medium only. Figure S3. Prediction errors and comparison of in silico results with experimental data using Synechocystis sp. PCC 6803. Table S1. Model parameters taken from Faizi et al. [15]●, Zavrel et al. [54]○ or estimated here. [file 12934_2019_1209_MOESM1_ESM.pdf]

Supplemental Material for the manuscript: "Optimal proteome allocation strategies for phototrophic growth in a light-limited chemostat" by M. Faizi and R. Steuer

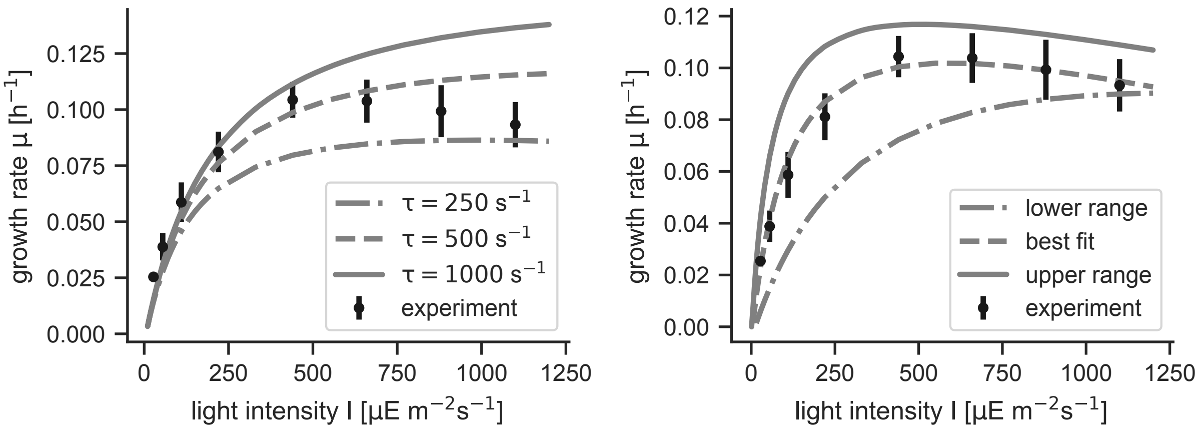

Figure S1: Parameter fitting. Optimized cellular growth rates μ for different turnover rates τ, $k_d = 10^{-7}$ and $\hat{\sigma} = 10$ nm^2 PSU^{-1} (left panel). Best fit for $\tau = 500$ s^{-1} was obtained with $k_d = 2.7 \cdot 10^{-7}$ and $\hat{\sigma} = 15$ nm^2 PSU^{-1} (right panel). Optimized growth rates for the lower range were simulated with $\tau = 500$ s^{-1}, $k_d = 5 \cdot 10^{-7}$ and $\hat{\sigma} = 5$ nm^2 PSU^{-1} and for the upper range with $\tau = 500$ s^{-1}, $k_d = 10^{-7}$ and $\hat{\sigma} = 30$ nm^2 PSU^{-1}.

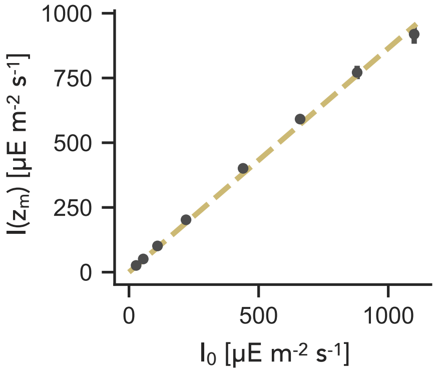

Figure S2: Light profile of photobioreactor filled with medium only. The background turbidity is fitted to $K_{bg} = 0.06$ cm^{-1}. Data points represent mean values of three photobioreactors. Data provided by T. Zavřel (personal communication).

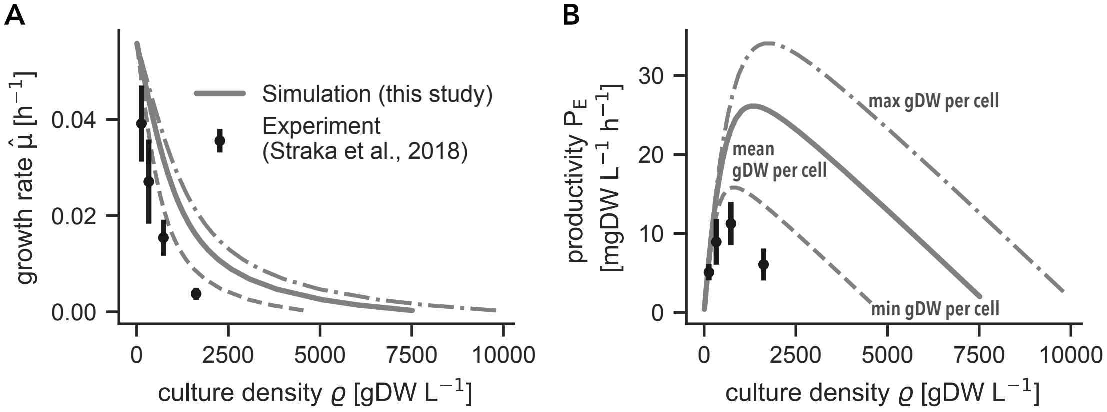

Figure S3: Prediction errors and comparison of *in silico* results with experimental data using *Synechocystis* sp. PCC 6803. Shown are predictions for the growth rate and the culture productivity for an incident light intensity $I_0 = 92$ μE m^{-2} s^{-1} (as used in Straka and Rittmann (2018)). Also shown are the error bars resulting from variance in the estimation of cellular dry weight (see section 'Model parametrization' in 'Methods'). Culture densities reported for conventional cultivation are typically significantly below the values suggested here. Shown is a comparison our results with the values reported by Straka and Rittmann (2018). While the functional form is in good agreement, the experimental and predicted values differ by a factor of 2.5. See section 'Discussion' for further analysis.

Table S1: Model parameters taken from Faizi et al. (2018)•, Zavřel et al. (2019)° or estimated here.

parameter	definition	value	source
V_{cell}	cell volume	$4.19 \cdot 10^{-15}$ [L cell^{-1}]	°
Proteins	protein mass per cell	$1.4 \cdot 10^{10}$ [aa cell^{-1}]	•
Quota	remaining cellular dry weight	10^{11} [carbon cell^{-1}]	
n_{ET}	transporter length	1681 [aa molecules^{-1}]	•
n_{EC}	length of carbon fixation enzyme	5400 [aa molecules^{-1}]	
n_{EQ}	enzyme length of quota catalyzing enzyme	23230 [aa molecules^{-1}]	
n_{EM}	length of metabolic enzyme complex	23230 [aa molecules^{-1}]	
n_R	ribosome length	7358 [aa molecules^{-1}]	•
n_{PSU}	length of photosynthetic unit	95451 [aa molecules^{-1}]	•
n_{PQ}	length of quota protein	300 [aa molecules^{-1}]	
$m_{c,ec}$	average carbon chain length of c_3	3	
$m_{c,em}$	amount of c_3 required for aa	2	
$m_{e,ec}$	amount of e consumed to create one c_3	23	
$m_{e,em}$	amount of e consumed to create one aa	22	
$m_{e,r}$	amount of e needed for one transl. elong. step	3	•
m_{hv}	photons required to activate one PSU	8	
m_{ϕ}	amount of e produced by PSU cycle	8	•
k^t_{cat}	maximal import rate	45360 [h^{-1}]	•
K_t	half-saturation constant of the transporter E_T	15 [μM]	•
k^c_{cat}	maximal carbon fixation rate	32700 [h^{-1}]	•
K_c	carbon fixation threshold	181 [μM]	•
k^q_{cat}, k^m_{cat}	average maximal turnover rate of an enzyme	72000 [h^{-1}]	
K_m, K_q	half-saturation constant of the metabolic enzymes E_M and E_Q	10000 [molecules cell^{-1}]	
γ_{max}	maximal translation rate	79200 [aa molecules^{-1} h^{-1}]	•
K_a, K_e	half-saturation constants for e and aa	10000 [molecules cell^{-1}]	•
d_P	protein degradation rate	0.043478 [h^{-1}]	°
k_{me}	energy maintenance rate	$7 \cdot 10^9$ [molecules cell^{-1} h^{-1}]	°
$\hat{\sigma}$	absorption cross section	15 [nm^2 PSU^{-1}]	
τ	maximal turnover rate of the photosynthetic unit PSU	1800000 [h^{-1}]	
k_d	rate constant for photodamage	$2.7 \cdot 10^{-7}$	
